# Supplementary material for: From Uncertainty to Consent: Educational Intervention Effects on Knowledge and Willingness to Donate Organs After Death
Source: Healthcare (Basel). 2025 Sep 30;13(19):2483. doi: 10.3390/healthcare13192483 (PMC12524708; doi:10.3390/healthcare13192483)
Supplement: Supplementary file 1 [file healthcare-13-02483-s001.zip › healthcare-3844716-supplementary.pdf]

Supplementary materials

Supplementary Table S1. Structure and Content of the Educational Lecture on Postmortem Organ Donation

Section	Content	Key Elements / Examples	Speaker(s)
Introduction	Opening remarks	Interactive survey, slogan "Real Talk on Organ Donation: Ask, Learn, Decide"	Moderator
Context	Current situation in Kazakhstan and globally	National waiting list, global imbalance in transplantation	Transplant coordinator
Myth-Fact Session	Common misconceptions vs. facts	7 myths addressed: (1) doctors won't save you if you consent, (2) brain death misdiagnosis, (3) black market, (4) limited survival after transplant, (5) "operate and forget," (6) financial reward for donation, and (7) body disfigurement	Coordinator & surgeon
System Overview	National transplantation system	Donor hospitals, transplant centers, HLA labs, air ambulance role	Transplant coordinator
Data & Legislation	National transplant activity and reforms	Trends in living vs. deceased donation, legislative amendments to National Health Code	Coordinator & surgeon
Personal Story	Patient perspective	Recipient perspective on life before and after transplantation	Transplant recipient
Closing & Call to Action	Post-lecture survey, encouragement to discuss at home	"One conversation can change a life," governmental donor registration (egov.kz) and information resources	Moderator

Supplementary Table S2. Comparison of baseline characteristics between participants who completed the post-test (n=97) and those lost to follow-up (n=32)

Variable	Post-test completed n(%) / M±SD	Post-test not completed n(%) / M±SD	χ^2 / t, p
School			
NU[1]	15 (53.6%)	13 (64.4%)	19.3, p<0.001
ENU[2]	28 (65.1%)	15 (34.9)	
AMU[3]	54 (93.1)	4 (6.9%)	
Gender			
Male	19 (76.0%)	6 (24.0%)	0.011, p=0.917
Female	78 (75.0%)	26 (25.0%)	
Age	18.58±1.69	19.63±3.10	2.423, p=0.017
Specialization			
Non-medical	29 (61.7%)	18 (38.3%)	7.22, p=0.007
Medical	68 (82.9%)	14 (17.1%)	
Language			
Kazakh	80 (80.0%)	20 (20.0%)	5.51, p=0.019
Russian	17 (58.6%)	12 (41.4%)	
Residence before university enrolling			
Rural	32 (82.1%)	7 (17.9%)	1.41, p=0.235
Urban	65 (72.2%)	25 (27.8%)	
Religion			
Islam	81 (76.4%)	25 (23.5%)	0.658, p=0.720
Agnosticism	5 (50.0%)	5 (50.0%)	
Atheism	11 (84.6%)	2 (15.4%)	
Religiosity (1-5)	2.86±1.15	3.03±1.03	0.770, p=0.443
Economic well-being (1-5)	3.57±0.88	3.56±0.80	0.026, p=0.979
Knowledge on organ donation (0-8)	5.34±1.67	5.16±2.24	0.494, p=0.622
Barriers of organ donation (1-5)	3.19±0.66	3.01±0.63	1.359, p=0.177
Attitudes Toward Postmortem Organ Donation			
LR[4]	20 (80.0%)	5 (20.0%)	1.87, p=0.392
DLCR[5]	50 (78.1%)	14 (21.9%)	
LC[6]	27 (67.5%)	13 (32.5%)	

[1] NU – Nazarbayev University, [2] ENU - L.N. Gumilyov Eurasian National University, [3] AMU – Astana Medical University, [4] LR – Lifetime Refusal, [5] DLCR – Decision Left to Close Relatives, [6] LC – Lifetime Consent.
